# Editorial Comment: Vaginoplasty tips and tricks

**DOI:** 10.1590/S1677-5538.IBJU.2020.0338.1

**Published:** 2021-02-03

**Authors:** Tiago Rosito

**Affiliations:** 1 Hospital de Clínicas de Porto Alegre - HCPA Porto Alegre RS Brasil Chefe do Serviço de Urologia, Hospital de Clínicas de Porto Alegre - HCPA, Porto Alegre, RS, Brasil.; 2 Universidade Federal do Rio Grande do Sul Hospital de Clínicas de Transgênero de Porto Alegre Brasil Cirurgião-chefe do Hospital de Clínicas de Transgênero de Porto Alegre da Universidade Federal do Rio Grande do Sul

## COMMENT

The paper by Santucci et al. (1) gave us a huge view of options in vaginoplasty for transgender care. The Crane center is one of the largest centers for transgender care in United States with great experience in this complex procedure. Gender-affirming surgery (GAS) is increasingly recognized as a therapeutic intervention and a medical necessity, with growing societal acceptance and this kind of knowledge is essential for urologists. It is therefore imperative to understand and establish best practice techniques for this patient population. The great problem with GAS is the poor quality of the studies about it. There is a lack of randomized trials to find the best way to treat and build a vagina. Most of the studies, including this by Santucci is based in personal experience. Some options as Skin mesh and buccal mucosa vaginoplasty, who achieve some good results in other centers were not described. As a whole the paper give some good tips and tricks for that surgeons that already do the procedure and opens a new world for the others,
